# Adenomatous polyposis coli-mediated control of β-catenin is essential for both chondrogenic and osteogenic differentiation of skeletal precursors

**DOI:** 10.1186/1471-213X-9-26

**Published:** 2009-04-08

**Authors:** Razvan L Miclea, Marcel Karperien, Cathy AJ Bosch, Geertje van der Horst, Martin A van der Valk, Tatsuya Kobayashi, Henry M Kronenberg, Georges Rawadi, Pinar Akçakaya, Clemens WGM Löwik, Riccardo Fodde, Jan Maarten Wit, Els C Robanus-Maandag

**Affiliations:** 1 Department of Pediatrics, Leiden University Medical Centre, Leiden, the Netherlands; 2 Department of Tissue Regeneration, Institute for Biomedical Technology, University of Twente, Enschede, the Netherlands; 3 Department of Human Genetics, LUMC, Leiden, the Netherlands; 4 Department of Urology, Leiden University Medical Centre, Leiden, the Netherlands; 5 Department of Animal Pathology, The Netherlands Cancer Institute, Amsterdam, the Netherlands; 6 Department of Medicine, Endocrine Unit, Massachusetts General Hospital, Harvard Medical School, Boston, Massachusetts, USA; 7 Galapagos, Romainville, 93230, France; 8 Department of Endocrinology and Metabolic Diseases, Leiden University Medical Centre, Leiden, the Netherlands; 9 Department of Pathology, Josephine Nefkens Institute, Erasmus Medical Centre, Rotterdam, the Netherlands

## Abstract

**Background:**

During skeletogenesis, protein levels of β-catenin in the canonical Wnt signaling pathway determine lineage commitment of skeletal precursor cells to osteoblasts and chondrocytes. Adenomatous polyposis coli (Apc) is a key controller of β-catenin turnover by down-regulating intracellular levels of β-catenin.

**Results:**

To investigate whether Apc is involved in lineage commitment of skeletal precursor cells, we generated conditional knockout mice lacking functional *Apc *in *Col2a1*-expressing cells. In contrast to other models in which an oncogenic variant of β-catenin was used, our approach resulted in the accumulation of wild type β-catenin protein due to functional loss of Apc. Conditional homozygous *Apc *mutant mice died perinatally showing greatly impaired skeletogenesis. All endochondral bones were misshaped and lacked structural integrity. Lack of functional Apc resulted in a pleiotropic skeletal cell phenotype. The majority of the precursor cells lacking *Apc *failed to differentiate into chondrocytes or osteoblasts. However, skeletal precursor cells in the proximal ribs were able to escape the noxious effect of functional loss of Apc resulting in formation of highly active osteoblasts. Inactivation of Apc in chondrocytes was associated with dedifferentiation of these cells.

**Conclusion:**

Our data indicate that a tight Apc-mediated control of β-catenin levels is essential for differentiation of skeletal precursors as well as for the maintenance of a chondrocytic phenotype in a spatio-temporal regulated manner.

## Background

During vertebrate embryogenesis, the axial and appendicular skeleton develop through endochondral bone formation. In this process, mesenchymal cells aggregate to form a chondrocytic template that prefigures the shape of the future bone. At the periphery of this cartilaginous mold, osteoblasts differentiate to form the bone collar. The cartilaginous mold is eventually replaced by bone in a step-wise program. Besides chondrocytes and osteoblasts, the skeleton also contains osteoclasts, which are of haematopoietic origin and play pivotal roles in both cartilage and bone resorption and remodelling [1-3].

Every step in the proliferation, differentiation, maturation, apoptosis, and resorption of both chondrocytes and osteoblasts is characterized by a specific transcriptional guideline [4]. Sox9, a high-mobility-group transcription factor, and Runx2, a Runt domain transcription factor, are both expressed in bi-potential skeletal precursor cells differentiating into either chondrocytes or osteoblasts [5-7]. Sox9 and Runx2 play leading roles in lineage commitment of these precursors: upregulation of Sox9 leads to chondrogenic differentiation [8], while activation of Runx2 is required for their osteogenic commitment [9].

Recently, based on mouse models, the canonical Wnt/β-catenin signaling pathway was found to act upstream of Sox9 and Runx2. In this pathway, in the absence of a Wnt signal, cytosolic β-catenin is degraded by the ubiquitination/proteasome system upon its phosphorylation at specific Ser-Thr residues by a destruction complex consisting of scaffold proteins such as Axin1, Axin2 (also known as Conductin) and the adenomatous polyposis coli (APC) tumor suppressor, and two kinases, namely glycogen synthase kinase 3β (GSK3β) and casein-kinase 1α (CK1α). Binding of Wnt to a complex composed of the transmembrane frizzled receptor and low-density lipoprotein receptor-related protein 5 or 6 (LRP5 or 6) co-receptor results in inactivation of the destruction complex and accumulation of cytoplasmic β-catenin. Upon its nuclear translocation, β-catenin acts as transcriptional co-activator in complex with transcription factors of the TCF/LEF family, leading to transcriptional activation of Wnt target genes [10]. In wild type mouse embryos, high levels of β-catenin and activation of canonical Wnt signaling have been found in osteoblastic precursors in developing skull and limb bones [11]. Accumulating evidence suggests that increased levels of canonical Wnt/β-catenin signaling inhibit Sox9 expression and activity, and stimulate Runx2 expression, leading to decreased chondrocyte differentiation and increased osteoblast differentiation, respectively [12-15]. Similar results have been found in transgenic mice with *Wnt14 *overexpression in *Collagen 2a1 *(*Col2a1*)-expressing cells [11].

It has been also demonstrated that β-catenin is required at an early stage to repress chondrocytic differentiation [15]. Upon conditional inactivation of *β-catenin *in the limb and head mesenchyme before or during early mesenchymal condensations, *Prx1*-expressing and *Dermo1*-expressing skeletal precursors, respectively, differentiate into chondrocytes instead of osteoblasts [11,15]. Finally, results on both constitutively active and inactivated β-catenin in *Osterix (Osx)*-, *Collagen 1a1 (Col1a1)*- or *Osteocalcin *(*Osc*)-expressing osteoblasts suggest that Wnt/β-catenin signaling coordinates bone formation by controlling the differentiation and activity of both osteoblasts and osteoclasts in a sequential, stage-specific manner [16,17].

Little is known about the mechanisms regulating β-catenin activity in skeletal precursors. Through its wide range of specific motifs and domains, APC is involved in multiple cellular processes such as signal transduction, cytoskeletal organization, apoptosis, cell adhesion and motility, cell fate determination, and chromosomal stability [18]. However, biochemical and genetic evidence has been provided showing that APC's main tumor suppressing activity resides in its ability to bind to β-catenin and induce its degradation, thereby acting as a strong negative regulator of the canonical Wnt pathway [19-21].

Familial adenomatous polyposis (FAP) patients heterozygous for an *APC *mutation frequently develop osteomas and dental anomalies [22]. Heterozygous *Apc*^1638N ^mutant mice occasionally develop osteomas (R. Fodde, personal communication). Homozygosity for the severely truncated *Apc*^Min ^and for the more hypomorphic *Apc*^1638N ^allele in the mouse results in a failure of primitive ectoderm development shortly after implantation, leading to lethality prior to gastrulation [23,24]. Mutant Apc disturbs the differentiation capacity of mouse embryonic stem (ES) cells in a quantitative and qualitative fashion depending on the dose of β-catenin signaling. Aberrant differentiation capacity of ES cells ranges from a strong differentiation blockade in case of two severely truncated *Apc*^Min ^alleles, to more specific neuroectodermal, dorsal mesodermal, and endodermal defects (e.g., no differentiation in bone or cartilage) in case of two hypomorphic *Apc*^1638N ^alleles [25,26]. Osteoblast-specific loss of Apc in the mouse leads to early onset of dramatically increased bone deposition and to lethality early in life [17]. However, Apc has not yet been linked with a role in the differentiation of skeletal precursor cells.

Here, we report that skeletal precursors of the axial and appendicular skeleton, when exposed to an uncontrolled rise of the β-catenin level due to conditional inactivation of *Apc*, lose their differentiation capacity to both the chondrogenic and osteogenic lineage. Moreover, conditional *Apc *mutant ribs show enhanced osteoblast activity, while the mutant nasal septum displays chondrocyte dedifferentiation. These results provide the first genetic evidence that Apc plays a crucial role throughout mouse skeletogenesis by regulating the differentiation of skeletal progenitor cells and maintenance of chondrocytes.

## Results

### Conditional Apc^15lox ^mice and transgenic Col2a1-Cre mice

Recently, we (ECR-M and RF) generated a novel mouse model carrying a conditional *Apc*^15lox ^allele where exon 15, encoding the majority of the coding region of Apc, and the polyadenylation signal, is flanked by lox*P *sites. Mice heterozygous and homozygous for the conditional *Apc*^15lox ^allele did not show any major abnormalities or susceptibility to tumors. *Apc^Δ15/+^*mice, heterozygous for the *Apc^Δ15^*mutant allele obtained by germline Cre-mediated deletion of exon 15, developed multiple intestinal tumors at an early age similar to* Apc^Min/+^*animals. These results indicate that Cre-mediated recombination of the *Apc*^15lox ^allele leads to inactivation of the Apc protein and to the constitutive activation of Wnt/β-catenin signaling (Robanus-Maandag et al., in preparation).

**Figure 1:**
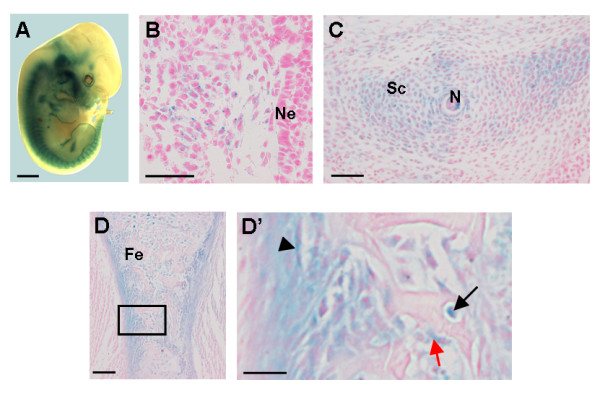
**Col2a1-*Cre*;Rosaflox mice express *Cre *at sites of endochondral bone formation**. (A-D) *LacZ *expression in Col2a1-*Cre*;Rosaflox embryos following *Cre *recombination, detected by whole-mount X-Gal staining. (A) Macroscopic picture of E12.5 Col2a1-Cre;Rosaflox embryo. (B) Transversal section of E9.5 embryo showing β-galactosidase-positive sclerotomal cells adjacent to the neural tube. (C) Transversal section of E12.5 embryo showing *LacZ *expression in vertebrae primordia. (D) Sagital section of E16.5 embryo showing LacZ expression in the femur. The boxed region in D is magnified in D' showing *LacZ *expression in the periosteum (arrow head), osteoblasts (red arrow) and osteocytes (black arrow). Ne, neuroepithelium; Sc, sclerotome; N, notochord; Fe, femur. Scale bars: 1 mm in A; 50 μm in B, D'; 100 μm in C, D.

Next, we investigated the temporal and spatial expression pattern of *Cre *in transgenic Col2a1-*Cre *mice [27] using *LacZ *reporter mice ("Rosaflox") [28]. Col2a1-*Cre*;Rosaflox embryos expressed *Cre *specifically at all sites of endochondral bone formation (Fig. 1A). In accordance with previous studies suggesting that *Col2a1 *is already expressed at E9.5 in the sclerotome of the somites [29], we detected *Cre *activity (based on positive LacZ staining) in mesenchymal condensations forming the sclerotome at E9.5 (Fig. 1B). At E12.5, LacZ-positive cells were identified in cartilage primordia later forming the vertebrae, long bones, sternum and cranial bones (Fig. 1C; data not shown). As reported in other Col2a1-*Cre *mouse lines [11,30], we found LacZ staining in the perichondrium at E14.5 (data not shown), and in the periosteum and primary spongiosa of long bones at E16.5, sites where osteoblasts normally differentiate (Fig. 1D, D'). The early onset (E9.5) of the *LacZ *expression in the sclerotome as well as its presence at later developmental stages (E14.5 and E16.5) in cells of the osteogenic lineage prompted us to conclude that the Col2a1-*Cre*-mediated recombination occurred in skeletal precursors characterized by both a chondrogenic and osteogenic differentiation potential.

### Heterozygous Apc^15lox/+ ^mice do not show any skeletal defect upon Col2a1-driven Cre expression

*Apc*^15lox/15lox ^mice were bred with Col2a1-*Cre *mice to generate conditional heterozygous Col2a1-*Cre*;*Apc*^15lox/+ ^mice. Microscopical analysis performed on Col2a1-*Cre*;*Apc*^15lox/+ ^and control *Apc*^15lox/+ ^embryos at various developmental stages (E12.5, E14.5, E16.5) displayed a normal spatio-temporal expression of all chondrogenic and osteogenic markers investigated (data not shown).

**Figure 2:**
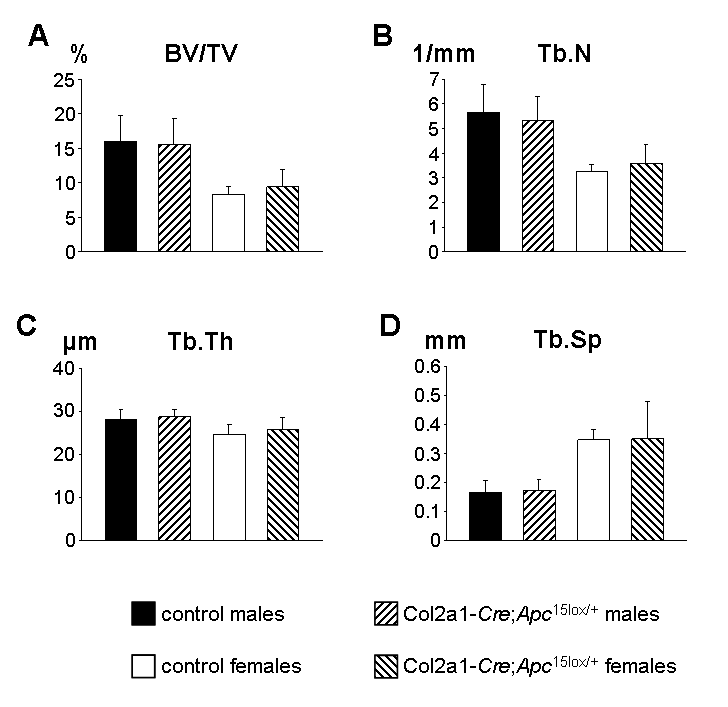
**Skeletal development occurs normally in Col2a1-*Cre*;*Apc*^15lox/+ ^mice**. (A-D) μCT analysis of the distal diaphysis of the femur did not reveal significant differences between 12-week-old Col2a1-*Cre*;*Apc*^15lox/+ ^mice and control littermates in any of the parameters investigated: (A) trabecular bone volume [BV/TV (%)], (B) number of trabeculae [Tb.N (1/mm)], (C) trabecular thickness [Tb.Th (μm)], and (D) trabecular separation [Tb.Sp (mm)]. All data represent mean values ± s.d

To study postnatal growth and bone acquisition, 18 Col2a1-*Cre*;*Apc*^15lox/+ ^mice (7 males, 11 females) and 11 *Apc*^15lox/+ ^mice (7 males, 4 females) were monitored for 12 weeks after birth. Mice of both genotypes were healthy, similar in appearance, size, body length/weight ratio and growth rate (data not shown). We next assessed bone architecture in these animals by micro-computed tomography (μCT) of the distal femora. No difference was detected between Col2a1-*Cre*;*Apc*^15lox/+ ^mice and gender-matched *Apc*^15lox/+ ^control littermates with respect to bone mineral density, trabecular bone volume fraction, trabecular number, trabecular thickness, and trabecular separation (Fig. 2A–D; data not shown). We further wanted to study whether conditional heterozygous *Apc *inactivation would lead to skeletal anomalies later in life. For this purpose, 10 Col2a1-*Cre*;*Apc*^15lox/+ ^mice (5 males and 5 females) and 5 *Apc*^15lox/+ ^male mice were followed for 24 months. At the end of this period, animals were sacrificed and tissues were analyzed microscopically using hematoxiline/eosine-stained sections. No important abnormalities could be distinguished in the skull, ribs, vertebral column and long bones. We evenly detected in both groups signs of cartilage degradation, fibrosis, and osteochondritis, pathological findings which most likely were all age-related (data not shown). Altogether, we considered conditional heterozygous *Apc *mutant embryos as controls for the next experiments.

### Homozygous Col2a1-Cre;Apc^15lox/15lox ^mice die perinatally due to severe defects in skeletogenesis

**Figure 3:**
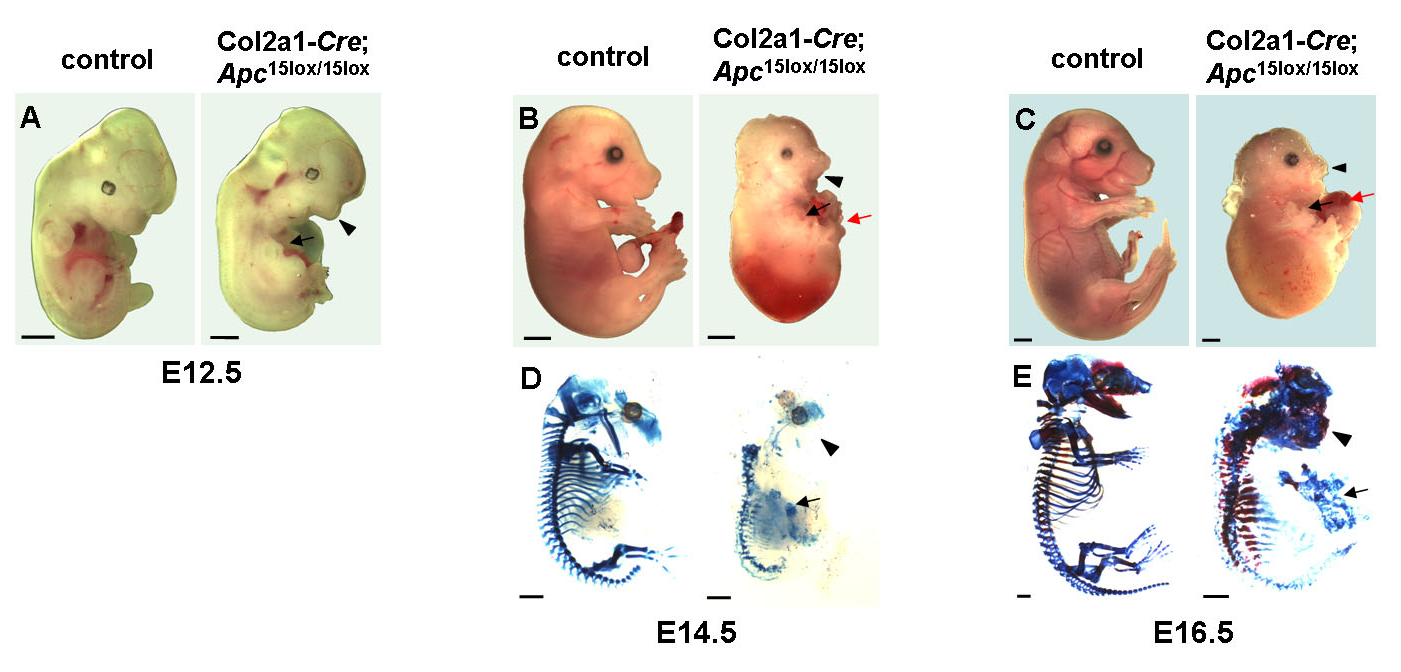
**Skeletogenesis is severely impaired in Col2a1-*Cre*;*Apc*^15lox/15lox ^embryos**. (A-E) Greatly impaired skeletal development and growth arrest of Col2a1-*Cre*;*Apc*^15lox/15lox ^embryos. Gross appearance (A-C) and Alcian blue and Alizarin red staining (D-E) of skeletal preparations of Col2a1-*Cre*;*Apc*^15lox/15lox ^embryos and control littermates at indicated developmental stages. Conditional *Apc *mutants showed lack of mandible outgrowth (arrowheads), poor limb development (black arrows), and an open thoracic and abdominal cavity (red arrows). Scale bars: 1 mm.

Col2a1-*Cre*;*Apc*^15lox/+ ^mice were crossed with *Apc*^15lox/15lox ^mice to generate conditional homozygous Col2a1-*Cre*;*Apc*^15lox/15lox ^mice (1:4). None of these mice were found at one month of age among 77 liveborn offspring. Of 27 dead pups found within the first month after delivery, only 5 pups on the day after delivery were Col2a1-*Cre*;*Apc*^15lox/15lox^. To further investigate the Col2a1-*Cre*;*Apc*^15lox/15lox ^phenotype, embryonic litters at various developmental stages were isolated. Eight of 31 embryos isolated between E16.5 and E19.5 were Col2a1-*Cre*;*Apc*^15lox/15lox ^(26%). We concluded that conditional homozygosity for this *Apc *mutant allele was perinatally lethal. At E12.5, Col2a1-*Cre*;*Apc*^15lox/15lox ^embryos, although normal in size, displayed poor mandible and limb outgrowth compared to control littermates (Fig. 3A). At E14.5 and E16.5, Col2a1-*Cre*;*Apc*^15lox/15lox ^embryos were much smaller in comparison to controls, displayed craniofacial abnormalities, short trunk, and an incomplete closure of both thoracic and abdominal cavities (Fig. 3B, C). Gross analysis further indicated a severe truncation of both upper and lower limbs. Already at E14.5, but more significantly at E16.5, Col2a1-*Cre*;*Apc*^15lox/15lox ^embryos presented large skin blisters especially in the dorso-lumbar region (Fig. 3C).

**Figure 4:**
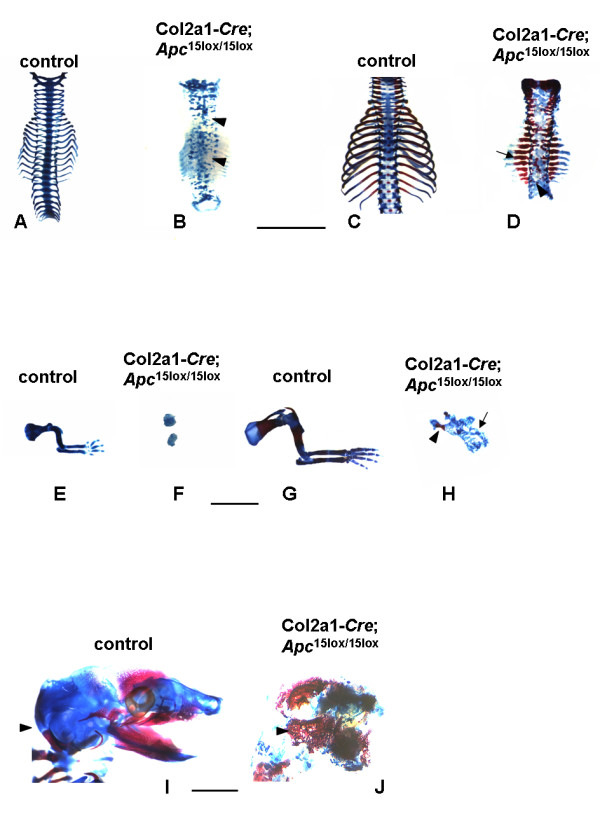
**Details of skeletal preparations**. (A-D) Vertebral column of control and mutant littermates at E14.5 (A, B) and E16.5 (C, D). Mutant vertebrae lacked structural integrity (arrowheads). At E16.5, mineralization was enhanced in the proximal part of the mutant rib (arrow). (E-H) Forelimb of control and mutant littermates at E14.5 (E, F) and E16.5 (G, H). At E16.5, only the scapula was identified (arrowhead), while more distal parts were represented by patchy cartilage aggregations (arrow). (I, J) Skull of control and mutant littermates at E16.5. The mutant displayed mineral deposition in the back skull corresponding to the cartilaginous structure in the control (arrowheads). Scale bars: 3 mm in A-H; 1 mm in I, J.

Skeletal preparations of mouse embryos stained with Alcian blue (chondrocyte matrix) and Alizarin red (mineralized matrix) of embryos at E14.5 revealed a clear difference in size between Col2a1-*Cre*;*Apc*^15lox/15lox ^mutants and control littermates (Fig. 3D). All mutant structures were severely misshaped and fragmented. Mutants failed to develop a cartilaginous mold of both the mandibles and the occipital bone. The axial skeleton contained patchy and irregular cartilaginous structures that did not organize in vertebrae. All 13 rib pairs could be individually distinguished, however, due to their inadequate orientation, size, and shape and due to lack of a sternum, no thoracic basket was formed (Fig. 4A, B). Distorted cartilage rudiments were found where forelimbs should normally arise (Fig. 4E, F), while no signs of bone formation were found in hindlimb rudiments. Furthermore, no cartilaginous primordia of pelvic bones were observed. Similar observations were made in Col2a1-*Cre*;*Apc*^15lox/15lox ^embryos at E16.5 (Fig. 3E). At this developmental stage however, distinct areas of mineralization were observed in most parts of the mutant skeleton. The mutant hind skull showed mineralized regions, whereas the control occipital and temporal bone primordias stained only with Alcian blue (Fig. 4I, J). Mutant proximal ribs in these Col2a1-*Cre*;*Apc*^15lox/15lox ^embryos were much thicker and shorter in comparison to those in control embryos, and stained intensively with Alizarin red (Fig. 4C, D). In the mutant forelimb, a hypoplastic scapula could be identified, whereas more distal components were agenetic and replaced by an irregular cartilaginous structure (Fig. 4G, H).

### Loss of functional Apc inhibits differentiation of skeletal precursors

Lack of functional Apc results in accumulation of cytoplasmic β-catenin, which subsequently translocates into the nucleus. This process can be well detected by immunohistochemistry (IHC). To investigate endochondral bone formation in Col2a1-*Cre*;*Apc*^15lox/15lox ^embryos, we analyzed vertebra formation at E12.5 and E14.5, and humerus development at E16.5 using IHC for β-catenin in combination with Alcian blue staining, and *in situ *hybridization (ISH) for several chondrocyte- and osteoblast-specific genes. Strongly elevated levels of β-catenin were seen at all sites of endochondral ossification in the Col2a1-*Cre*;*Apc*^15lox/15lox ^embryos at E12.5, E14.5 and E16.5, indicating efficient Col2a1-*Cre*-mediated *Apc *inactivation.

**Figure 5:**
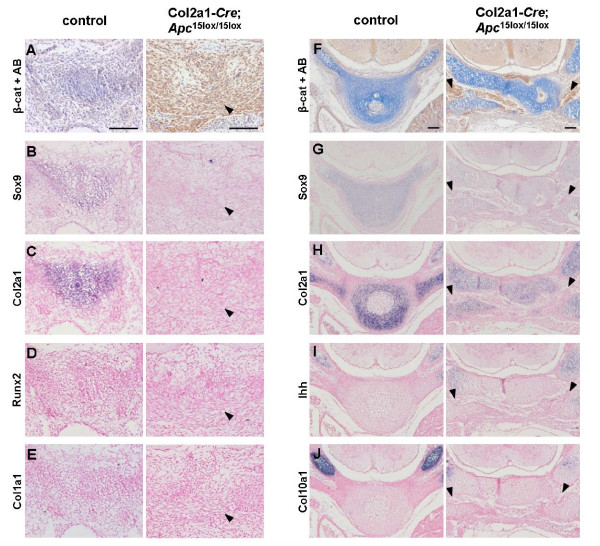
**Abnormal axial skeleton formation of Col2a1-*Cre*;*Apc*^15lox/15lox ^embryos already detectable at E12.5**. (A) Immunostaining for β-catenin combined with Alcian blue (AB) staining, and (B-E) gene expression analysis by in situ hybridization with indicated probes on consecutive transversal sections of the sclerotome of a Col2a1-*Cre*;*Apc*^15lox/15lox ^embryo and control littermate at E12.5. (F-J) Similar analysis of vertebrae primordia at E14.5. β-Catenin-positive spindle-shaped cells lacked expression of all indicated chondrogenic and osteogenic markers (arrowheads). Scale bars: 100 μm.

At E12.5, transversal sections of control vertebral primordia showed normal mesenchymal cell condensation and subsequent chondrogenic differentiation (Fig. 5A–C). Chondrocytes stained negatively for β-catenin, started to deposit an Alcian blue-stained matrix, and expressed the nascent chondrocyte markers *Sox9 *and *Col2a1*. In marked contrast, mutant sclerotomal cells failed to condense into skeletal primordias. They showed strong nuclear β-catenin staining and displayed a mesenchymal-like spindle shape morphology. These cells expressed neither *Sox9*, nor *Col2a1*, implying that conditional loss of functional Apc in skeletal precursors inhibited mesenchymal cell condensation and chondrogenic differentiation. Next, we investigated whether these cells had switched their commitment to the osteogenic lineage due to the accumulation of β-catenin. Surprisingly, they did not express the early osteoblast markers *Runx2 *and *Col1a1*, suggesting that β-catenin accumulation due to *Apc *inactivation impaired osteogenic differentiation of skeletal precursors as well (Fig. 5D, E).

At E14.5, chondrocytes in the control vertebrae did not stain positively for β-catenin, displayed an intensely Alcian blue-stained matrix and expressed both early (*Sox9*, *Col2a1*) and mature chondrocyte markers, like *Indian hedgehog *(*Ihh*) and *Collagen 10a1 *(*Col10a1*), indicating a normal progression of endochondral ossification (Fig. 5F–J). Although somite formation was present, mutant vertebrae were heavily crumbled and failed to organize in a cartilaginous anlage. Occasionally Alcian blue-positive clusters of chondrocytes were seen, which lacked detectable β-catenin immunostaining and were positive for chondrogenic marker expression. These cells were probably derived from non-recombined cells due to mosaicism of *Cre *expression. Surrounding these cartilage islands, mesenchymal-like spindle-shaped cells were observed. Comparable to the defects observed at E12.5, these cells expressed high levels of nuclear β-catenin due to *Apc *inactivation and lacked not only an Alcian blue-positive matrix but also expression of both chondrogenic and osteogenic markers (Fig. 5F–J; data not shown).

**Figure 6:**
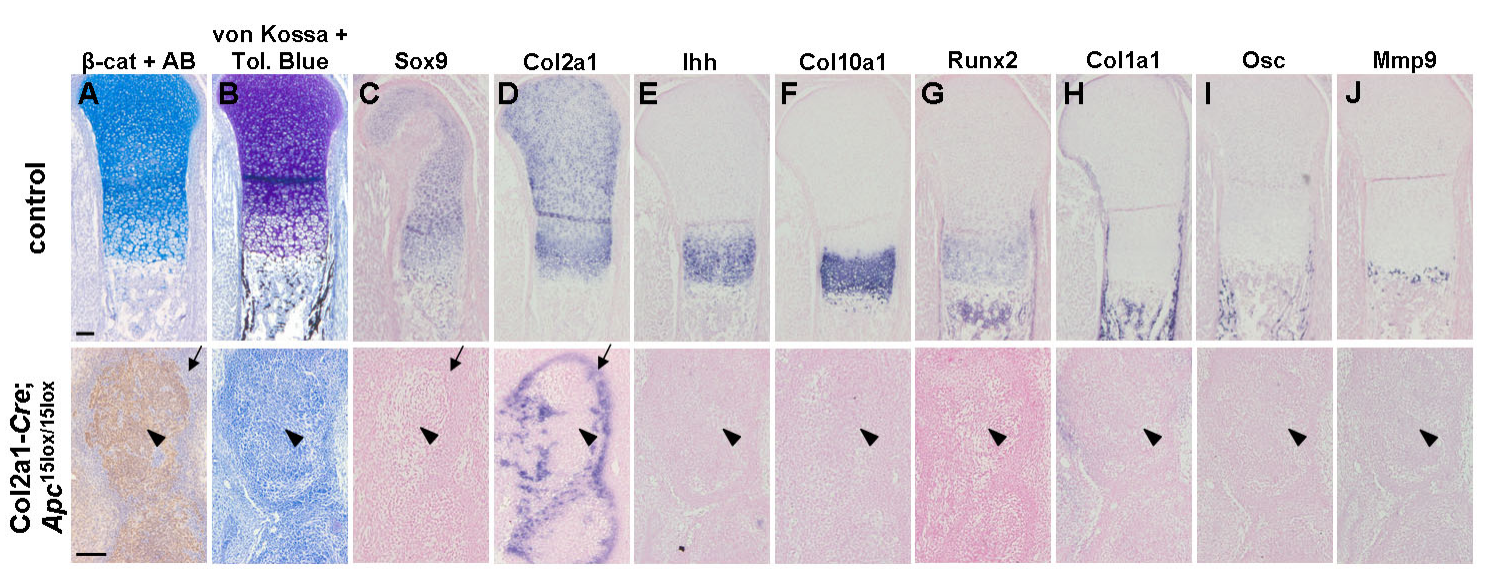
**No chondrogenic and osteogenic differentiation in the developing humerus due to lack of functional *Apc***. (A-B) Immunostaining for β-catenin combined with Alcian blue (AB) staining (A), combined von Kossa-Toluidine blue staining (B), and (C-J) gene expression analysis by in situ hybridization with indicated probes for (C-F) chondrocytes, (G-I) osteoblasts and (J) osteoclasts on consecutive transversal sections of the developing humerus of a Col2a1-*Cre*;*Apc*^15lox/15lox ^embryo and control littermate at E16.5. β-Catenin-positive spindle-shaped cells organized in clusters and failed to express chondrogenic and osteogenic markers (arrowheads). β-Catenin-negative cells at the periphery of these clusters expressed early chondrogenic markers only (arrows), probably due to lack of Cre-mediated loss of functional *Apc*. Scale bars: 100 μm.

At E16.5, chondrocytes of control proximal humeri did not express detectable β-catenin protein levels and were surrounded by a proteoglycan-rich matrix, which stained positively with both Alcian blue and Toluidine blue (Fig. 6A, B). They were organized in growth plates with a characteristic spatial expression pattern of the chondrogenic markers *Sox9*, *Col2a1*, *Ihh*, and *Col10a1 *(Fig. 6C–F). Young osteoblasts in the perichondrium, periosteum and primary spongiosa were surrounded by a mineralized osteoid as detected by von Kossa staining (Fig. 6B) and expressed *Runx2 *and *Col1a1 *(Fig. 6G, H). Mature osteoblasts expressed *Osc *(Fig. 6I), while osteoclasts expressed Matrix metalloproteinase 9 (*Mmp9*) (Fig. 6J). In contrast, mutant humeri were completely misshaped and contained nuclear β-catenin-positive cells that were organized in clusters, showing a mesenchymal-like shape (Fig. 6A). Similar to our observations at E12.5 and E14.5, these cells expressed neither chondrogenic, nor osteogenic markers (Fig. 6C–I). In addition, no *Mmp9 *expression could be detected (Fig. 6J), suggesting that differentiation of bone-resorbing cells was impaired as well. These β-catenin-positive cell clusters were surrounded by chondrocytes expressing *Sox9 *and *Col2a *and lacked positive staining for β-catenin. These cell most likely have not undergone a recombination event as observed at E14.5 (Fig. 6A, C, D).

### Increased osteoblastogenesis in proximal ribs of Col2a1-Cre;Apc^15lox/15lox ^embryos

**Figure 7:**
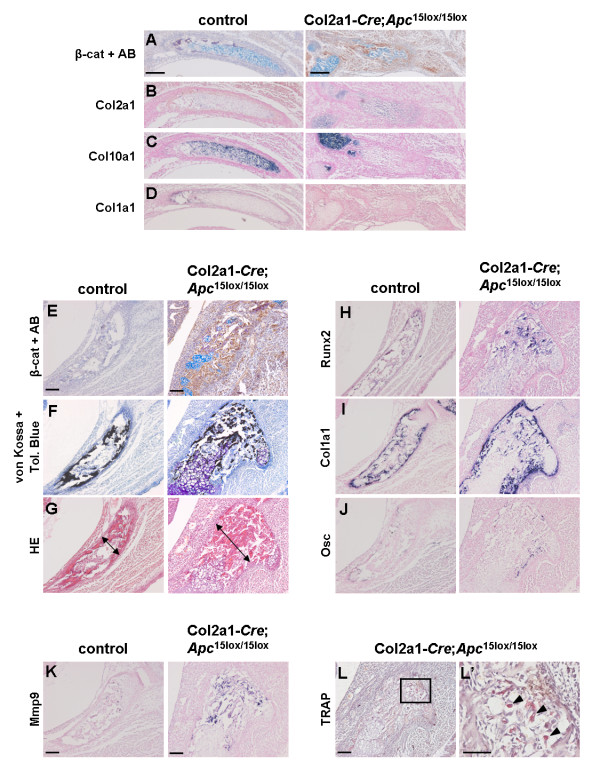
**Conditional *Apc *inactivation enhances osteoblast formation and mineral deposition in the developing proximal rib**. (A-L) Immunostaining for β-catenin combined with Alcian blue (AB) staining (A, E), combined von Kossa-Toluidine blue staining (F), hematoxylin/eosin staining (G), gene expression analysis by in situ hybridization with indicated probes for chondrogenic (B, C), osteogenic (D, H-J) and osteoclastogenic differentiation (K) on consecutive transversal sections of the developing proximal rib of a Col2a1-*Cre*;*Apc*^15lox/15lox ^embryo and control littermate at E14.5 (A-D) and E16.5 (E-K). The double-headed arrows in G indicate the thickness of the rib. (L) Tartrate-resistant acid phosphatase (TRAP) staining of the developing proximal rib of a Col2a1-*Cre*;*Apc*^15lox/15lox ^embryo at E16.5. The boxed region in L is magnified in L' showing multinucleated osteoclasts (arrowheads) staining positive for TRAP. Scale bars: 100 μm in A-L; 50 μm in L'.

Despite the inhibitory effect of *Apc *inactivation on differentiation of skeletal precursors in long bones and vertebrae, proximal ribs of Col2a1-*Cre*;*Apc*^15lox/15lox ^embryos at E16.5 showed clearly enhanced mineralization upon skeletal staining (Fig. 4D). Therefore, we analyzed the development of these skeletal structures in more detail. The ribs develop through endochondral ossification from the paired lateral sclerotomic areas [31]. Formation of the proximal rib depends on the notochord and the ventral neural tube, whereas development of the distal part depends on the surface ectoderm [32]. At E14.5, proximal ribs of control embryos were cartilaginous and contained mature chondrocytes that did not stain for β-catenin (Fig. 7A–C). Mutant proximal ribs were severely misshaped and contained β-catenin negatively stained cartilage islands, accounting for the positive Alcian blue staining observed upon skeletal preparation (Fig. 4B, 7A). β-Catenin-positive cells were negative for chondrogenic and osteogenic markers (Fig. 7A–D). At E16.5, the β-catenin-negative proximal ribs of control embryos consisted of cartilage and mineralized bone matrix as indicated by combined von Kossa-Toluidine blue staining (Fig. 7E, F; data not shown). They contained chondrocytes, osteoblasts, and osteoclasts as assessed by ISH (Fig. 7H–K; data not shown). In contrast, proximal ribs of mutant littermates stained strongly positive for β-catenin and were significantly thicker and shorter compared to those of control embryos (Fig. 4C, D and 7E–G). They consisted of a massive mineralized bone matrix and a poorly developed bone marrow cavity, although osteoclast differentiation and activity were normal as assessed by ISH for *Mmp9 *and TRAP staining, respectively (Fig. 7K, L, L'). Interestingly, β-catenin-positive cells expressed all osteogenic markers analyzed (*Runx2*, *Col1a1*, and *Osc*), indicating that, unlike in the long bones and vertebrae, *Apc *inactivation in skeletal precursors of the proximal ribs did not impair osteoblastogenesis (Fig. 7H–J). Since Ihh is a critical regulator of osteoblastogenesis, we subsequently tested whether the increased ossification might be due to increased *Ihh *expression in the non-recombined neighbouring chondrocytes. The β-catenin-negative cells, however, matured normally expressing all chondrocyte markers investigated (*Sox9, Col2a1, Ihh and Col10a1*) at similar levels compared to control cartilage (data not shown). The abundant presence of a bone matrix combined with evidence of functional osteoclasts suggested that the β-catenin-positive osteoblasts were sclerotic.

### Chondrocyte dedifferentiation in the nasal septum of Col2a1-Cre;Apc^15lox/15lox ^embryos

**Figure 8:**
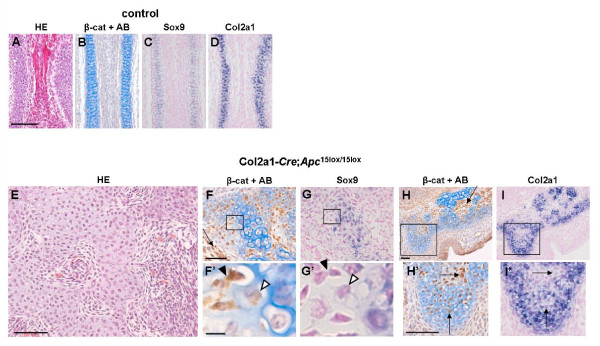
**Dedifferentiation in the nasal septum of Col2a1-*Cre*;*Apc*^15lox/15lox ^embryos at E16.5**. (A, E) Hematoxylin/eosin staining, (B, F, H) immunostaining for β-catenin combined with Alcian blue (AB) staining, and (C-D, G, I) gene expression analysis by in situ hybridization with indicated probes for chondrogenic differentiation on consecutive transversal sections of the developing nasal septum of a Col2a1-*Cre*;*Apc*^15lox/15lox ^embryo and control littermate at E16.5. (F', G', H', I') High magnification pictures of the boxed regions in F, G, H, and I, respectively. Mesenchymal-like β-catenin-positive cells (arrow in F, H) were present between crumbled cartilage islands. Within these cartilage islands, although displaying chondrocytic morphology and an Alcian blue stained matrix, most of the β-catenin-positive cells did not express *Sox9 *(arrowheads in G') or *Col2a1 *(arrows in I'). Scale bars: 100 μm in A, E, F, H; 5 μm in F', H'.

The nasal septum is a midline vertical plate of hyaline cartilage, which undergoes endochondral ossification in postnatal life [33]. Endochondral ossification of the caudal and dorsal borders of the septum, when combined with interstitial expansion, has the effect of displacing the facial skeleton away from the neurocranium and thus enlarging the skull [34]. At E16.5, chondrocytes forming the nasal septum of control mice did not stain for β-catenin, were surrounded by an Alcian blue-positive matrix, and expressed *Sox9 *and *Col2a1 *(Fig. 8A–D). In the mutant nasal cartilage we distinguished crumbled chondrogenic islands surrounded by β-catenin-positive cells with an undifferentiated mesenchymal-like phenotype (Fig. 8F–H). The chondrogenic islands consisted of round cells embedded in chondrons surrounded by extracellular matrix (ECM) (Fig. 8E). Interestingly, molecular analysis of these chondrogenic islands revealed the presence of two cell populations: β-catenin-negative and β-catenin-positive cells. The former expressed chondrogenic markers like *Sox9 *and *Col2a1*, and their ECM stained positive with Alcian blue, whereas the latter did not express any chondrogenic or osteogenic markers, while their ECM stained significantly less with Alcian blue (Fig. 8F–I; data not shown).

The presence of β-catenin-positive cells in the chondrogenic islands suggested that these cells, due to mosaicism of *Cre *expression, had initiated normal chondrocyte differentiation before undergoing *Apc *inactivation. Subsequently, the increased level of β-catenin triggered the loss of expression of the early chondrogenic markers and initiated degradation of the ECM. These observations were indicative of dedifferentiated chondrocytes. Similar observations were made in cartilaginous rudiments at other sites of endochondral bone formation, but the effect was most pronounced in the nasal septum (data not shown).

## Discussion

### Conditional homozygous loss of functional Apc severely disrupts mouse skeletogenesis via stabilized β-catenin

According to most of the transgenic mouse studies reported, levels of β-catenin, the effector of the canonical Wnt ligands, need to be downregulated in skeletal precursor cells to enable chondrogenic differentiation, whereas elevated β-catenin levels promote differentiation into osteoblasts [11,12,15,35,36]. This theory is partly based on observations in heterozygous gain-of-function models in which Cre-mediated recombination results in the expression of oncogenic β-catenin. The cellular mechanisms controlling the biological effects of oncogenic β-catenin in the presence of wild type β-catenin are largely unknown. In addition, there are no reports on the role of Apc in regulation of skeletal precursor differentiation via control of β-catenin in the mouse. Here, we have focused on this important role of the multifunctional protein Apc, binding to and downregulating β-catenin. We have selectively inactivated one or both alleles of *Apc *in murine *Col2a1*-expressing cells. Our data indicate that the *Col2a1 *promoter is suitable for this study, since Cre-mediated recombination starts very early (E9.5) in skeletal precursor cells that have not yet committed to the chondrogenic or the osteogenic lineage, consistent with previous findings in other Col2a1-*Cre *lines [11,30].

Conditional heterozygous inactivation of *Apc *does not result in a detectable level of its target β-catenin as determined by IHC. Moreover, heterozygous Col2a1-*Cre*-mediated *Apc *inactivation does not interfere with embryonic skeletal development, postnatal growth or bone acquisition up to 24 months of age, as determined by histological and μCT analysis. Our data imply that the level of Apc protein produced by a single functional *Apc *allele is sufficient to mediate appropriate β-catenin degradation. This is in agreement with normal body weight, size, and growth of young Apc^Min/+ ^mice [37].

In marked contrast, conditional inactivation of *Apc *results in a strongly elevated level of (wild type) β-catenin in skeletal precursors, leading to greatly impaired embryogenesis and perinatal lethality. The significantly reduced size and the vast range of skeletal malformations in these embryos is most likely due to the specific Col2a1-*Cre *activity in skeletal primordias at a very early embryonic stage starting at E9.5 resulting in massive β-catenin accumulation in the developing endochondral skeleton. Probably several factors, like the open rib cage and the severe malformation, from E14.5 on have led to the perinatal lethality. The loss of the multiple β-catenin-independent functions of the Apc protein might have contributed to the gravity and complexity of the skeletal phenotype observed in Col2a1-*Cre*;*Apc*^15lox/15lox ^mice as well [18]. Moreover, since *Col2a1 *expression is not completely restricted to skeletal tissues during mouse embryogenesis [29], we can not exclude that the severity of the phenotype might have been partly due to loss of functional *Apc *in other Col2a1-*Cre*-expressing cell types.

### Apc is crucial for both chondrogenic and osteogenic differentiation of skeletal precursors

Wnt/β-catenin signaling represents a mechanism in mesenchymal precursor cells for selecting between chondrocytic and osteoblastic fates. This key regulating role in lineage commitment has been attributed to β-catenin. Indeed, conditional gain-of-function mutation of *β-catenin *leads to decreased chondrocyte differentiation in *Prx1*-expressing and *Col2a1*-expressing cells [12,15]. However, corresponding increased osteoblast differentiation has not been observed in these models, instead, a decreased osteoblast marker expression has been seen in case of *Prx1*-expressing cells, suggesting that activation of β-catenin negatively affects skeletogenesis [12,15]. In addition, conditional loss-of-function mutation of *β-catenin *in *Prx1*-expressing cells leads to increased expression of not only chondrocyte but also early osteoblast markers [15]. These data strongly suggest that β-catenin negatively regulates the differentiation of mesenchymal cells into a common skeletal precursor [38].

We report here that in the vast majority of endochondral skeletal elements, precursor cells lacking functional Apc express strong nuclear β-catenin staining and fail to differentiate into both chondrogenic and osteogenic lineages. These data are in line with the inability of mouse embryonic stem cells carrying specific bi-allelic *Apc *mutations to differentiate into bone and cartilage [25]. Our data are also consistent with those based on conditional stabilization of β-catenin in mesenchymal skeletal precursors which had an undifferentiated appearance [15]. This consistency strongly suggests that, notwithstanding the multiple functions of Apc, its β-catenin-controlling role is the most important during skeletogenesis. We conclude that Apc plays a crucial role in differentiation of skeletal precursors in vertebrae and long bones: it enables the differentiation into both skeletal lineages by decreasing the level of β-catenin.

### Loss of functional Apc in skeletal precursors of the proximal rib stimulates osteogenesis

Although in the vast majority of the endochondral skeleton both chondrogenic and osteogenic differentiation is inhibited due to loss of functional *Apc *in skeletal precursors, we find a different phenotype in the proximal ribs. Notwithstanding the cartilaginous structure at E14.5, proximal ribs of Col2a1-*Cre*;*Apc*^15lox/15lox ^mutants at E16.5 show abundant bone matrix deposited by osteoblasts, invariably expressing high levels of nuclear β-catenin. Since osteoblasts do not express *Col2a1*, these cells are most likely derived from *Col2a1*-expressing skeletal precursors lacking functional Apc. This implies that, in contrast to other skeletal elements, skeletal precursors of the proximal ribs are able to escape from the noxious effects of strongly elevated β-catenin levels on differentiation of precursor cells by an as yet unknown mechanism. Since *Ihh *expression is normal in the non-recombined neighbouring chondrocytes, we speculate that Ihh may be a prime target for inducing osteoblastogenesis in the recombined precursor cells counteracting the noxious effect of β-catenin.

Despite the evidence of functional osteoclasts, the intensely ossified proximal ribs show a strongly diminished bone marrow cavity, rendering it likely that the increased bone formation is due to osteosclerosis. These observations are in agreement with other data, showing that enhanced canonical Wnt signaling can increase bone mass through stimulation of osteoblast activity rather than inhibition of osteoclast formation and activity [39-41]. Such an osteopetrotic phenotype has only been seen in mice with conditional loss of functional *Apc *or constitutively active β-catenin in already differentiated osteoblasts, resulting in dramatically increased bone deposition [17,42].

### Functional Apc is required to maintain the chondrocyte phenotype

We have found clear evidence for the occurrence of chondrocyte dedifferentiation due to β-catenin accumulation in the nasal septum. Morphologically characterized chondrocytes, which were nuclear β-catenin-positive, lacked expression of typical chondrocyte markers. Furthermore, they were imbedded in an ECM containing significantly less proteoglycans.

Given the noxious effect of increased β-catenin levels on chondrocyte formation (our data and [12,15]), these cells most likely have undergone Cre-mediated loss of functional *Apc *after completion of the initial stages of chondrocyte differentiation. Mouse models with an increased level of β-catenin in *Col2a1*-expressing cells show accelerated chondrocyte maturation [11,12]. We have found no indication for this phenomenon, implying that the high level of β-catenin due to loss of *Apc *does not result in chondrocyte maturation but in chondrocyte dedifferentiation. Our data suggest that accumulated β-catenin triggers this dedifferentiation program not only through inhibition of chondrogenic marker expression but also by enhancing the loss of ECM presumably through stimulation of matrix-degrading enzymes. It has been demonstrated that β-catenin increases expression and activity of a number of enzymes involved in matrix degradation [43-45]. β-Catenin stabilization has been associated with dedifferentiation of articular chondrocytes in vitro upon serial monolayer culture, or treatment with retinoic acid or IL1β [46]. Dedifferentiated chondrocytes have also been observed at other sites of endochondral bone formation in the Col2a1-*Cre*;*Apc*^15lox/15lox ^embryos, however, the presence of these cells was most pronounced in the nasal septum. Altogether, our data indicate that Apc is required to suppress β-catenin for maintenance of the chondrocytic phenotype.

## Conclusion

We show here for the first time that Apc, by negatively controlling the levels of β-catenin, is a critical regulator of the differentiation of skeletal progenitor cells. Conditional inactivation of the mouse *Apc *gene results in a heterogeneous skeletal phenotype. Based on our results, we postulate that Apc-mediated control of the dosage of transcriptionally active β-catenin protein is directive for the differentiation program of skeletal precursor cells. In the vast majority of the skeletal precursors, loss of functional *Apc *leads to a strongly increased β-catenin level, resulting in the formation of an undifferentiated mesenchymal cell, which lacks differentiation potential for both osteogenic and chondrogenic lineages. When the inhibitory effect of a strongly increased β-catenin level in the skeletal precursors is reduced, highly active osteoblasts arise. Strong repression of β-catenin in these precursors is required for chondrogenesis. Support for our hypothesis on the importance of the dosage of Apc and β-catenin is provided by observations in Col2a1-*Wnt14 *transgenic mice [11]. Higher levels of *Wnt14 *expression resulting in a high level of β-catenin block differentiation of skeletal precursors into chondrocytes or osteoblasts, whereas lower levels of *Wnt14 *expression result in enhanced ossification. We provide evidence that Apc plays a crucial role in modulating the β-catenin level during mouse skeletogenesis in a spatio-temporal regulated manner. In skeletal precursor cells, Apc is required for differentiation into both chondrocytes and osteoblasts. In addition, Apc is essential in chondrocytes to maintain their phenotype and enable their maturation.

## Methods

### Transgenic mice

All animal studies were approved by the ethical committee of the Leiden University Medical Centre and complied with national laws relating to the conduct of animal experiments. The *Apc*^15lox/+ ^mouse was generated by gene targeting in IB10 embryonic stem cells, using a 22.5 kb targeting vector containing lox*P *sites flanking the last exon of *Apc*, i.e. exon 15. Lox*P *sites were inserted in the *Bgl*II site of intron 14 and in the *Apa*I site approximately 350 bp downstream of the *Apc *polyadenylation signal. Exon 15 of the *Apc *gene encodes for codons 660 to 2842 of the Apc protein and harbours all the functional domains of Apc involved in β-catenin regulation as well as the C-terminal domains binding to microtubules, DLG, and EB1. Therefore, following Cre-mediated deletion of exon 15, functionality of the remaining protein will be fully impaired with respect to the main function of Apc, i.e. β-catenin regulating. Moreover, as deletion of exon 15 also removes the polyadenylation signal, no stable mRNA is produced and as a consequence no stable truncated protein will be generated. A full description of this new conditional *Apc *mouse model is currently in preparation (Robanus-Maandag et al., in preparation). Col2a1-*Cre *mice [27] were mated with *Apc*^15lox/15lox ^mice. Of the offspring, Col2a1-*Cre*^+/-^;*Apc*^15lox/+ ^mice were mated with *Apc*^15lox/15lox ^mice to obtain Col2a1-*Cre*^+/-^;*Apc*^15lox/15lox ^mice. *LacZ *reporter mice were obtained from Dr. Xiaohong Mao [28]. Routine mouse genotyping was performed on tail DNAs by PCR (Robanus-Maandag et al., in preparation).

### Skeletal analysis

Skeletons of mouse embryos were stained with Alcian blue and Alizarin red for cartilaginous and mineralized tissues, respectively, according to standard procedures [47]. For micro-computed tomography (μCT) analysis, femora were recovered from 12-week-old mice after death and processed as described [48].

### β-galactosidase staining, Histology, Immunohistochemistry, In Situ Hybridization

Whole mount β-galactosidase staining was performed as described [49], from E16.5 on after removal of the skin. For histology, immunohistochemistry, and in situ hybridization, specimens were fixed in phosphate-buffered formalin, embedded in paraffin, and sectioned at 6 μm. Hematoxylin/eosin, Nuclear red, Toluidine blue, and von Kossa stainings were performed according to standard procedures. For immunohistochemistry, sections were treated with 1% H_2_O_2 _in 40% methanol/60% TBS for 30 minutes to reduce endogenous peroxidase activity. For antigen retrieval the sections were boiled in Tris-EDTA pH 9.0 for 20 minutes. Blocking was performed with 5% blocking buffer for 30 minutes at 37°C (Boehringer Ingelheim). Sections were incubated with the primary mouse monoclonal antibody against β-catenin (1:100; BD Transduction Laboratories) overnight at 4°C, followed by incubation with the second antibody biotin-conjugated rabbit anti-mouse IgG (1:300; Amersham Biosciences) for 45 minutes at 37°C. The biotinylated proteins were detected by incubation with horseradish peroxidase-conjugated streptavidin (1:200; Amersham Biosciences) for 30 minutes at 37°C and visualized with DAB (Sigma). After counterstaining with Alcian blue for 15 minutes and hematoxylin for 1 minute, sections were dehydrated and embedded in Histomount (BDH). For in situ hybridization, digoxigenin-labeled single-stranded RNA probes were prepared using a DIG RNA labeling kit (Boehringer) following the manufacturers' instructions. All probes are available upon request. In situ hybridization was carried out as described [15,50]. Images were taken with a DXM-1200 digital camera (Nikon).

## Authors' contributions

The studies were designed and initiated by MK as principal investigator, with the help of RF and ECR-M; Col2a1-*Cre *mice were provided by TK and HMK; mutant mice were generated and genotyped by CAJB and ECR-M; embryo experimental work and analysis were performed by RLM; μCT analysis was performed by GR; data interpretation was carried out by RLM assisted by MK, GH, MAV, PA, CWGML, RF, JMW, and ECR-M; the manuscript was written by RLM with the assistance of all co-authors. All authors read and approved the final manuscript.
